# Development of a Visualisation Approach for Analysing Incipient and Clinically Unrecorded Enamel Fissure Caries Using Laser-Induced Contrast Imaging, MicroRaman Spectroscopy and Biomimetic Composites: A Pilot Study

**DOI:** 10.3390/jimaging8050137

**Published:** 2022-05-13

**Authors:** Pavel Seredin, Dmitry Goloshchapov, Vladimir Kashkarov, Anna Emelyanova, Nikita Buylov, Yuri Ippolitov, Tatiana Prutskij

**Affiliations:** 1 Solid State Physics and Nanostructures Department, Voronezh State University, University Sq.1, 394018 Voronezh, Russia; goloshchapovdl@gmail.com (D.G.); kash@phys.vsu.ru (V.K.); anna.liviero238@mail.ru (A.E.); buylov@phys.vsu.ru (N.B.); 2 Scientific and Educational Center, Nanomaterials and Nanotechnologies, Ural Federal University, Mir Av., 620002 Yekaterinburg, Russia; 3 Department of Pediatric Dentistry with Orthodontia, Voronezh State Medical University, Studentcheskaya St. 11, 394006 Voronezh, Russia; dsvgma@mail.ru; 4 Sciences Institute, Autonomous University of Puebla (BUAP), Puebla 72570, Mexico; tatiana.prutskij@correo.buap.mx

**Keywords:** a laser-induced contrast visualisation, initial caries, microRaman spectroscopy, biomimetic composites

## Abstract

This pilot study presents a practical approach to detecting and visualising the initial forms of caries that are not clinically registered. The use of a laser-induced contrast visualisation (LICV) technique was shown to provide detection of the originating caries based on the separation of emissions from sound tissue, areas with destroyed tissue and regions of bacterial invasion. Adding microRaman spectroscopy to the measuring system enables reliable detection of the transformation of the organic–mineral component in the dental tissue and the spread of bacterial microflora in the affected region. Further laboratory and clinical studies of the comprehensive use of LICV and microRaman spectroscopy enable data extension on the application of this approach for accurate determination of the boundaries in the changed dental tissue as a result of initial caries. The obtained data has the potential to develop an effective preventive medical diagnostic approach and as a result, further personalised medical treatment can be specified.

## 1. Introduction

Owing to improvements in biomimetic material sciences [1,2,3,4], new forms of replacement dentin and dental enamel have been developed. In accordance with the biomimetic concept, minimally invasive intervention is used during the operative treatment of caries lesions [5]. The treatment of the lesions is based on the excision of tissue affected by caries but with the maximum preservation of the part of tissue that was not affected by the caries attack and its consequent demineralisation. For a long time, visual examinations combined with radiography have been the key diagnostic methods for the originating caries; however, this is already insufficient for comprehensive and timely aid [6].

The intensive development of ultrasound diagnostics [7], optical tomography [8], magnetic resonance [9], reflectance confocal microscopy [10,11] and fluorescence [12] enabled a new level of caries diagnoses, the determination of stages in the development of this disease and also the enamel remineralisation [13]. However, a wide spread of caries in the occlusive fossae and fissures requires new methods of online screening [14].

Although some modern achievements in therapeutic dentistry have shown progress in this area [15], early and precise diagnostics of carious lesions remains a top priority. Spatial spectroscopic visualisation [16] with a high resolution within the regions of caries development at the micro- and nanoscale is a key challenge in the search for new methods in caries prevention and treatment [17,18].

A special case in the visualisation of carious microregions is when the disease penetrates through fissures in the enamel and further into dentin tissue, thus developing very intensively. The solution of this problem can be found through the difference in the physical properties of the intact and affected dental tissues. The intact region of a tooth and the region with the beginnings of a carious lesion can be compared and visualised with the use of structural and spectroscopic methods of analysis, based on the high sensitivity of the proposed techniques [19,20]. The information obtained from these methods makes it possible to describe not only the crystal and chemical characteristics of the apatite that comprises the dental tissue but also the chemical transformations in the organic component of teeth [21]. An important feature of these spectroscopic methods is their ability to determine the bacterial status of the detected area and the state of the biofilm [22,23,24].

At present, to detect carious lesions in the initial stage of development, various visualisation methods are widely employed, e.g., light and laser fluorescence and autofluorescence [12,25,26,27,28,29]. These methods enable differentiation of the intact and carious dental tissue, which demonstrate different levels of sensitivity [12,14,29]. The main principle is to use low-power laser emissions to scan the dental tissue to detect carious lesions [30]. The use of different wavelengths can detect differences in the density, structure and chemical composition of the biological tissue, while fluorescence losses are appropriate for assessing the stage and depth of the caries infection [12,24]. Currently, approaches to caries visualisation based on the fluorescence of the intact and carious tissue assist dentists in providing minimally invasive treatment; moreover, they can do so without X-ray analysis [28,30,31,32,33], which can be inefficient, particularly when it is impossible to diagnose an incipient lesion and the destruction of tooth enamel in fissure system [26,30,33].

Some recent works have demonstrated an unprecedented combination of resolution and contrast during caries visualisation in vitro, i.e., with the use of synchrotron microtomography [16]. However, applying such approaches in clinical practice can be complicated and sometimes even inappropriate. Visualisation, sensitivity and rapid testing of diagnostic instruments are the main applicability criteria for diagnostic methods. Therefore, the overwhelming majority of modern investigations are concerned with the use of Raman spectroscopy for clinical applications in dental diagnostics [34,35,36]. Raman spectroscopy can be considered the gold standard for the analysis of the mineralisation of the hard dental tissues [21,34,35,36]. The basic approach to diagnosing caries with Raman spectroscopy is connected with detecting changes in chemical composition through the difference in the spectral characteristics of the hard dental tissues [37,38]. This method has been applied in dental investigations as a noncontact analytical technology for in vivo chemical specification of the intact and carious dentin [37,39,40,41].

Early screening of caries with Raman spectroscopy has been realised by employing the characteristic signatures of molecular vibrational spectroscopy, such as the position of the combination scattering band, the full width at half maximum (FWHM) and the shape of the band profile (as a measure of the crystal lattice disorder [19,35,42]. Many conclusions concerning the condition of dental tissue have been made based on differences in the orientation of the mineral crystallites (mean value) and the texture distribution determined from the Raman spectra [34,43]. This approach enables the association of the mechanical properties of dental enamel with its chemical composition and ensures good sensitivity [44]. Moreover, phosphate mapping has also been used as a diagnostic technique; maps can be rendered based on the PO_4_^3−^ Raman mode at 960 cm^−1^ [21,35,45]. In this case, the depth of the lesion can be determined based on the variations of the intensity of the phosphate peak [21,45,46,47]. A further approach is based on evaluating the ratio of the Amide I and PO_4_^3−^ Raman peaks [48,49]. This diagnostic method has the potential for improving the biochemical assessment of carious dentin using a nondestructive technique [36].

However, when determining the boundaries of the microregion in the dental tissue affected by the initial stage of caries, it seems inappropriate to focus only on the transformation of the mineral and organic components of the dental matrix. In order to exclude the subjective factor, it is important to analyse not only the affected matrix area but also the regions of bacterial lesions in the tissue [50,51]. Therefore, successful screening and visualisation of the incipient caries should be based on the overall assessment of the laser-induced fluorescence and Raman microspectroscopy data [52].

The advantages of simultaneously employing fluorescence and Raman microspectroscopy to detect caries development in teeth have been demonstrated in [25,40,52]. However, these reports did not involve comparing and analysing the light-scattering and autofluorescence of dental tissues at the initial stages of the pathology used to visualise the boundaries of carious lesion microregions and the applicability of such a diagnostic instrument in clinical practice. Our current work is concerned with these problems.

## 2. Materials and Methods of Investigation

In our work, five groups of teeth samples from patients aged 18–25 were investigated. Human teeth with different degrees of carious lesions were collected in compliance with the Declaration of Helsinki and the requirements of the ethical protocol. The samples were extracted from the patients in accordance with orthodontic indications or due to the complicated carious process. Following collection, the samples were immediately placed into distilled water and kept at +4 °C.

**Figure 1:**
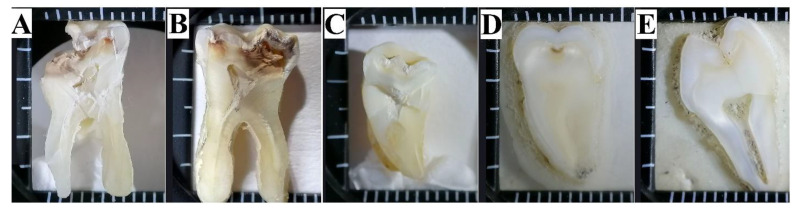
Optical images of the human teeth samples studied in this work. (**A**–**E**)-Sample Group name (see Table 1).

Segments of teeth were prepared for examination. The samples of teeth were separated into segments using a low-speed (200 rpm) water-cooled diamond blade [38,40] in such a way that the cross section included the area of the fissure system. Figure 1 presents typical images of the collected samples under 5× magnification, while their description is given in Table 1. Five samples of each type were prepared. Classification of the carious lesions is provided in accordance with the International Caries Detection and Assessment System (ICDAS) [53].

**Figure 2:**
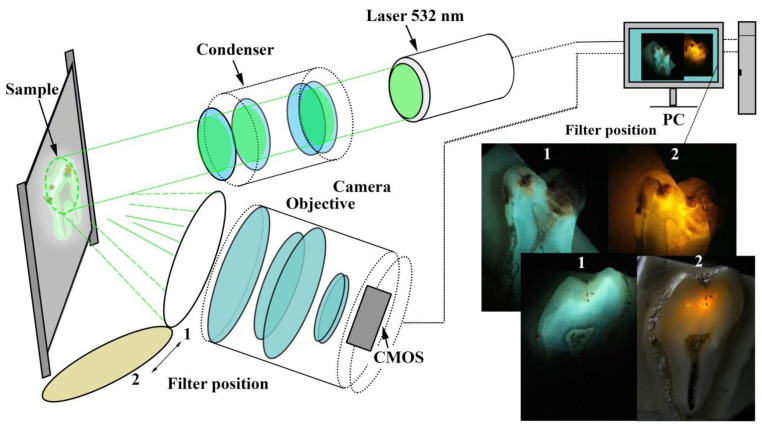
Schematic image of the apparatus for observing laser-induced fluorescence.

Different defects in the apatite structure, the nanocrystal sizes and chemical transformations in apatite affect the spectral position and profile of lines in Raman scattering [19,38,54]. For this reason, a biomimetic composite was obtained that was used as a reference sample; it was designed to simulate the properties of enamel. The biocomposite was created from carbonate-substituted hydroxyapatite (CHAP) with a CO_3_ percentage of ~1.5%. The CHAP was prepared using the wet-chemistry technique of titrating a concentrated solution of calcium hydroxide with 0.3 M orthophosphoric acid (H_3_PO_4_). The calcium hydroxide was preliminarily obtained by thermal annealing of avian eggshells [55]. To reproduce the amino acid matrix of enamel and dentin, the main polar amino acids of the tooth matrix were used (L-arginine hydrochloride (C_6_H_15_ClN_4_O_2_), L-histidine and L-lysine hydrochloride (C_6_H_15_ClN_2_O_2_)). The ratio of organic and mineral components was 5:95 to reproduce the properties of dental enamel. The prepared slices and samples of biomimetic composites were analysed with a laser-induced fluorescence apparatus (see the scheme in Figure 2) and noncontact microRaman and luminescence spectroscopy.

The laser-induced contrast imaging setup (see Figure 2) was designed to visualise the caries-induced changes in the tooth tissue. The cut section of a tooth was illuminated by the parallel beam of a solid-state laser with a wavelength of 532 nm. The 532 notch filter (TECHSPEC, Edmund Optics, NJ, USA) was installed and used to block the reflected laser light, and thus, after filtering, the obtained image contained only the fluorescent emission from the tooth surface. An excitation light intensity of approximately 10 mW was used for all measurements. The signal reflected by the sample was collected and registered with a Canon EOS camera with the Canon lens EF and a CMOS-sensor (Tokyo, Japan).

Raman and luminescence spectra were obtained with a Raman-luminescence microscope RamMix M532 (EnSpectr, Russia) combined with an Olympus optical microscope (Shinjuku, Tokyo, Japan). The study was performed with excitation laser radiation with a wavelength of 532 nm. The power at the surface was 30 mW, and the diameter of the analysed area was about 2 μm. The choice of the microregions on the surface of a sample was realised using an automated motorised 2-axis stage providing minimum step-by-step shifts of 300 nm. A signal from the surface of a sample was collected with a 60× objective. The area of the analysed microregion was 2 × 2 μm. The spectral data were processed, and the baseline was corrected in Origin 8 program suite.

The results of the preliminary analysis of the data showed that the Raman and luminescence spectra within chosen group of the samples contained a similar set of modes, which differ in intensity at different scanning points. Therefore, for the purpose of clarity, the Raman and luminescence spectra presented below in the work and obtained from microareas of healthy and carious teeth samples were averaged in groups.

## 3. Results

### 3.1. Laser-Induced Fluorescence

**Figure 3:**
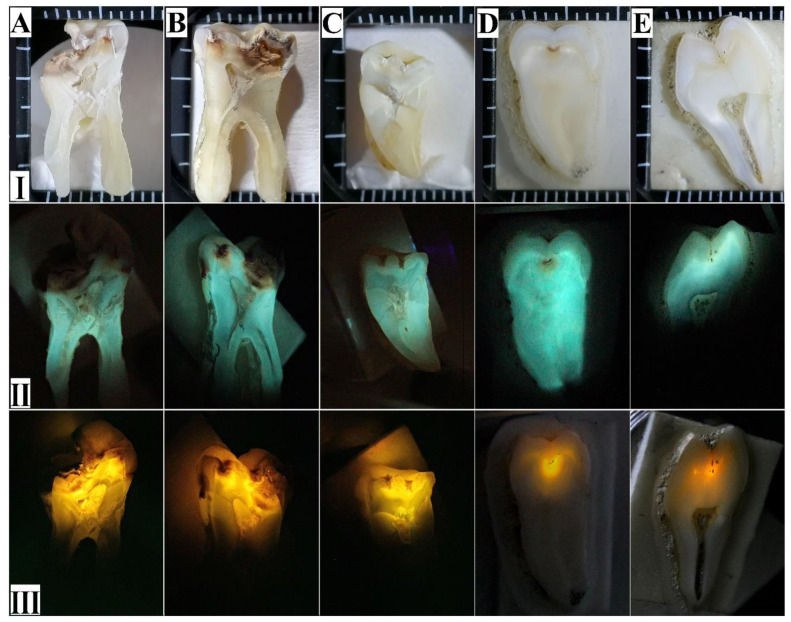
Images of the dental tissue samples with different degrees of development of the carious process: Line **I**—optical images; Line **II**—images of the reflectance signal obtained using the laser-induced fluorescence technique; Line **III**—images obtained using the laser-induced fluorescence technique after applying a notch filter. (**A**–**E**)-Sample Group name (see Table 1).

Figure 3 (line I) presents the optical images of the dental tissue samples with different degrees of development of the carious process (see Table 1), together with the contrast images obtained with the use of laser-induced fluorescence technique (line II). In order to enhance the contrast, an external light source was not used, and both the reflected (scattered) signal from the sample (see Figure 3, line II) and the signal obtained after the inclusion of a notch filter (Figure 3, line III) were recorded.

The destructive lesions of the dental tissue in samples A–C (ICDAS 6–3) and the incipient caries in sample E (ICDAS 1) can be visually detected even without laser-induced contrast visualisation. Analysis of the optical images of sample E (ICDAS 1) obtained at a magnification up to 5× demonstrated with a high degree of certainty that the detection of carious microlesions (incipient caries), even when the slice progresses through the system of fissures, is visually impossible.

Comparison of the optical images of the samples and their fluorescence shows that the intensity and colour of the luminescence for the intact and caries-affected dental tissue are different. The regions of intact dental tissues in the images obtained in reflectance (Figure 3, line II) do not noticeably differ in colour and demonstrate maximal luminosity. The destructive carious lesions of enamel and dentin, along with the macro- and microvoids, are visualised as dark areas in the images.

The difference in the contrast of the distinct regions in the images enables determining the boundaries of bulk defects in the dental tissues. The contrast of the images obtained from the reflected signal (Figure 3, line II) is the sum of the contributions of the autofluorescence for the intact and carious tissues and the laser-induced emission of the affected tissues. While considering the region of the carious lesions in samples A, B and C, graduation in the colour scale can be seen. At the same time, the contrast decreases in sample D (ICDAS 2), while for sample E (ICDAS 1), no difference in the contrast is observed, thus indicating the necessity of additional filters while analysing the initial carious lesions.

The images obtained by using laser-induced fluorescence with a notch filter and analysis of the spatial contrast in the images (Figure 3, line III) demonstrate that the process of caries development (i.e., the stage of the lesion) can be easily visualised. Complex analysis of the images in Figure 3 demonstrates that the fluorescence intensity separated by the notch filter is considerably higher in the regions surrounding the destroyed dental tissue. It is well known that under the laser-induced fluorescence at 532 nm, the emission bands of porphyrins (associated with the bacteria resulting in caries) can be stimulated [12,24,25,56,57]. In this case, the intense fluorescence detected in the red spectral range [12,57,58] indicates not only an increase in the microorganism content in the area of contrast but also implies possible disorganisation processes in the dental tissue, i.e., the changes are already occurring in the organic components of enamel and dentin in the investigated teeth (Figure 3, line III).

### 3.2. MicroRaman Spectroscopy

**Figure 4:**
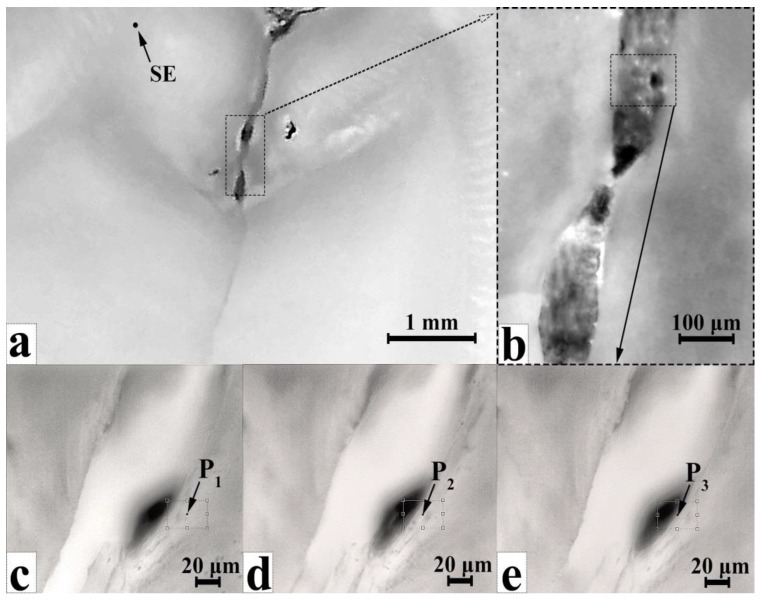
Optical images of regions of sample E with incipient caries (ICDAS 1): (**a**) 8× magnification; (**b**) area of the carious cavity in the enamel, 130× magnification; (**c**–**e**) images of microregions in the carious region involving microcavities, showing microRaman scanning points (200× magnification).

Owing to the small size of the microregions of the incipient carious lesions of dental enamel, their clinical recognition by visual inspection is impossible [30,34]. Therefore, to implement precise and the earliest possible detection of carious processes, noncontact Raman spectroscopy can be efficiently employed to differentiate the microscopic areas of the enamel and carious lesions with high spatial resolution. Figure 4a shows a region in sample E, specifically the region of the demineralised lateral wall of the fissure at different magnifications (Figure 4a—8×; Figure 4b—130×; Figure 4c–e—200×).

**Figure 5:**
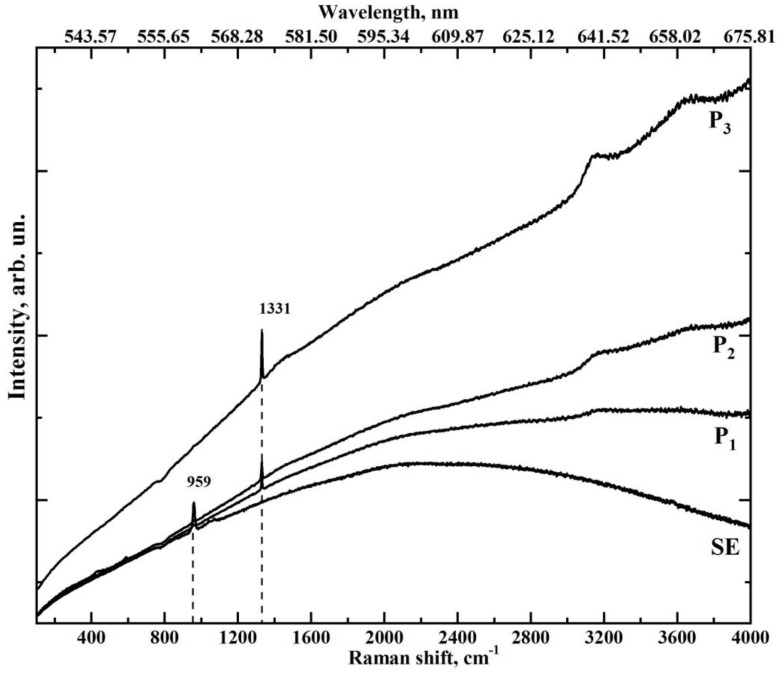
Results of microRaman spectroscopy of the different microregions of sample E (demineralisation of the lateral wall of the fissure, incipient caries in the fissure, ICDAS 1) obtained with the use of the automated motorised double-axis stage.

**Figure 6:**
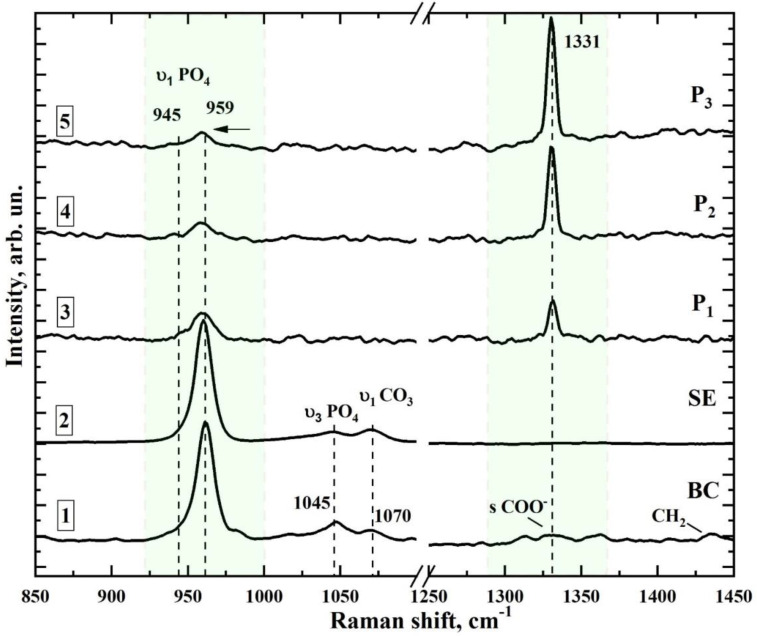
Results of microRaman spectroscopy of the different microregions of sample E (demineralisation of the lateral wall of the fissure, incipient caries in the fissure, ICDAS 1) after fluorescence background correction.

The typical microRaman scattering spectra with the fluorescence background obtained from the regions containing sound enamel (region SE in Figure 4a) and different regions of the enamel with incipient caries at 10, 5 and 0.5 μm from the edge of the cavity (Figure 4c–e, respectively) are presented in Figure 5. In Figure 6, we present the same Raman spectra after fluorescence background correction in two spectral regions 850–1075 and 1250–1450 cm^−1^. Additionally, Figure 6 shows Raman spectrum of the biomimetic composite (BC).

Comparison of the spectra of the BC that simulates properties of the dental enamel and the natural enamel SE (curves 1 and 2 in Figure 6, respectively) shows that the spectrum of the composite reproduces the features of the spectrum of natural enamel very well, suggesting the bioinspired material itself can be applied in vitro.

The analysis of the results shows that the most intensive mode in the Raman spectra of the sound enamel at SE and bioinspired material at BC is the characteristic vibration of the phosphate ion υ_1_ PO_4_^3−^ in carbonate-substituted calcium hydroxyapatite, which is the base mineral component of native dental tissue and the biomimetic composite [21,42,59]. The mode of υ_1_ PO_4_^3−^ is characterised by the specific position and the FWHM, together with the features in the spectra of natural tissues [60]. For example, the υ_1_ PO_4_^3−^ mode in the spectra is localised near 959.7 cm^−1^, coinciding with the reference data [21,35,42,43], and with its position in the spectrum of the biomimetic composite (see Figure 6). This result represents the crystal–chemical characteristics of carbonate-substituted apatite, namely, the characteristic atomic ratio Ca/P and the carbonate ion (CO_3_^2−^) content in the apatite crystal lattice in the natural enamel (B-type substitution [60]).

Using the automated motorised double-axis stage, Raman microscanning was performed near the carious cavity of sample E with increasing distance from the boundary of the carious microlesion. This enables detection of the boundaries of the area in the enamel characterised by the demineralisation processes. These regions can be found by the change in position, intensity and shape of the Raman band of PO_4_^3−^ in the spectral range 945–959 cm^−1^, relative to its characteristics in the spectrum of sound enamel (Figure 6) according to the known reference data [42,59].

Figure 5 shows that disorganisation in the crystal structure of the dental enamel apatite occurring due to the development of the carious process results, not only in broadening of the phosphate ion band but also in a decrease in its intensity while approaching the boundary of the carious microcavity (Figure 4c–e) from the intact tissue zone. An additional maximum in near 945 cm^−1^ can be attributed to the weak (transition) phosphates [22,59,61,62,63,64] and to the amorphised apatite-like structure [40,61,64].

While approaching the region of enamel changed by the carious process, one more characteristic maximum at about 1330 cm^−1^ can be detected in the Raman spectra. The intensity of this maximum increases considerably at the boundary of the carious cavity and the sound enamel. In Raman spectroscopy, this maximum is associated with the presence of bacterial microflora [40,65].

**Figure 7:**
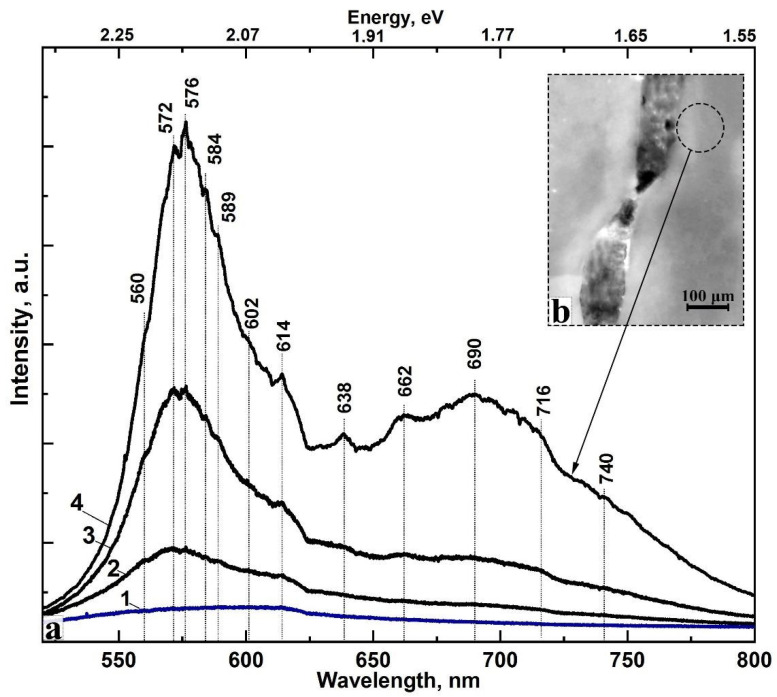
Luminescence spectra (**a**) and the micro area view where spectra collected (**b**). (1)—sound enamel from the point SE (Figure 4a); (2)—point P_1_; (3)—point P_2_; (4)—point P_3_, located 0.5 μm from the edge of the cavity (Figure 4e).

With the use of a Raman-luminescence microscope registering luminescence spectra from the points P_1_, P_2_ and P_3_, located at a distance of 10, 5 and 0.5 μm from the edge of the cavity, respectively (Figure 4c–e), the characteristic autofluorescence spectra of the sample were registered (Figure 6). The luminescence spectra were recorded using the notch filter included in the setup. Additionally, Figure 7 represents the spectrum of the sound enamel from the point SE for comparison (see Figure 4a). It can be easily seen that the spectrum of sound enamel (ICDAS 0) demonstrates a single broad maximum. Note, that the luminescence spectra of sound enamel demonstrate much less intensity as compared with the spectrum from the area near the carious microlesion, which coincides with the known data from literature [25].

Luminescence spectrum at the point P_3_, located 0.5 μm from the edge of the carious cavity, has two broad maxima in the yellow-green and red range of the spectrum (575 and 685 nm) and several less intense characteristic features are seen. Analysis of our results and their comparison with the already known reference data demonstrates that the appearance of features in the luminescence spectra at 572, 576, 638, 690 and 716 nm is associated with the light emission of porphyrins—products of the vital activity of microorganisms (bacteria causing caries) [25,31,56,66]. If the fluorescence is excited with the laser, applying lasers with different wavelengths in rather wide interval (405, 532, 630 and 740 nm), emission bands of porphyrins can be observed in the red spectral range, and they only slightly differ by a significant shift of the main peaks [12,31,57,58,67,68]. The source of light excitation with the wavelength of 532 nm resulted in appearance of the emission bands in the range 500–750 nm characteristic for bacteria fluorophores and the products of their activity. Therefore, it becomes clear that the shape of the fluorescence band obtained from the microregions of dental enamel is mainly affected by the processes related to the caries development and the level of its formation in the area analysed.

The observed ratio of the peak intensities at 575 and 685 nm corresponds to the degree of development of the carious process (ICDAS 1–2), which is determined from the convolution of the reference spectra characterising these stages [69].

The spectrum at point P_2_ is characterised by the same features that are present in the spectrum at point P_3_ (Figure 7). While moving away from the edge of the carious cavity, intensity of the bands in the spectrum is considerably reduced. Moreover, intensity of the peaks at 575 and 685 nm is appreciably redistributed, thus indicating a decrease in part of the porphyrins. This result correlates well with the Raman spectroscopy data (Figure 6). As for point P_1_ that is most distant from the edge of the carious cavity, its spectrum is much less intensive than the one at point P_2_. Note, that in fact, no low-intensive features are observed in this spectrum as compared with the spectrum at point P_3_.

We want to note that the fluorescence background of Raman spectra (Figure 5) is of the same character as in the luminescence spectra from every microregion (see Figure 7). One should also note that in the Raman scattering spectra before the correction for the fluorescence background, obtained in the condition of a long-term photobleaching, there appear luminescence bands in the range 640 and 660 nm (Figure 5). Maximums of these bands, in their turn, are present in the luminescence spectra as well (Figure 7), while the intensity of the other features is appreciably reduced.

The Raman-luminescent mapping of the near-boundary areas adjacent to the affected region (Figure 4c–e) demonstrates that penetration of the vital activity products of the bacterial microflora into the tissue can be detected about ~50 μm^2^ around the carious microlesion, observed from the gradual loss of intensity for the band at 1330 cm^−1^ (Figure 6).

It should be noted that in spite of the fact that Raman spectra and luminescence were obtained using one and the same setup their simultaneous demonstration seems to be inappropriate since the intensity of Raman scattering is much less than the signal of luminescence. As a result, the acquisition interval of the Raman signal in our experiment was considerably higher than that one for the luminescence signals. Moreover, due to the long acquisition time for the Raman signal and photobleaching of the sample, it is impossible to observe a desired signal in the decreasing luminescence background. In addition, notice that for the late stages of caries development in dental enamel (ICDAS 3–6), local destruction of dental tissue is observed (Figure 3, series I), owing to the presence of gaps in the enamel, which explains why the fluorescence spectrum of the enamel in the regions of ICDAS 2 is less useful for the task of detecting early caries.

## 4. Discussion

The analysis of the results of investigating samples of dental tissue with different degrees of carious lesions (1–6 ICDAS), using laser-induced contrast visualisation (LICV) and microRaman scattering, enabled an understanding of the correspondence between the regions of the fluorescent images of the samples and the changes in the mineral and organic components of the dental tissue under caries attack, accompanied by the spread of bacterial microflora.

Using a wavelength in the green spectral range for the excitation provides deeper penetration into a tooth and thus enhances the qualitative and quantitative analysis of the signal emitted by the dental tissue. For both deep and incipient carious lesions, LICV with the notch filter allows the precise separation of the regions in the hard dental tissue undergoing changes. Visualisation of the carious lesions obtained without the filter shows that for the deep carious lesions, anatomically different areas provide certain fluorescence. There is also a specific gradation of colours in the images that allows the visualisation of the bacterial invasion. This agrees with the spectral analysis data and known works that have demonstrated the different contributions of porphyrins and microorganisms producing these substances at the different stages of caries [12,24,25,56,57]. At the same time, for the initial stages of caries (ICDAS 1–2), the use of the visualisation mode without a filter application, that is, observation of the reflected signal, is less informative (Figure 3, line II). The application of the notch filter enables the detection of microregions by the release of autofluorescence associated with a bacterial lesion of the dental tissue (Figure 3, line III).

The incipient stage of caries (ICDAS 1–2) requires additional analysis of the near-surface layers, where maximal contrast is observed using LICV. This task can be successfully completed by introducing Raman microspectroscopy into the exploratory scheme. Indeed, Raman scattering, which has a spatial resolution of several micrometers, enables the determination of local variations in the structure of the enamel connected with the developing carious process. These variations are mediated not only by the changes in the crystal structure of the apatite but also by the penetration of the vital activity products formed by bacterial microflora (Figure 6, curves 3–5) into the dental tissue. This phenomenon is supported by analysis of the profiles of the registered emission spectra (Figure 7) from the region adjacent to the caries microlesion. Different products of bacteria in the oral cavity, including porphyrins and pentosidine, provide a characteristic set of bands in the spectrum of infected dental tissue [25]. The observed spectral emission profile demonstrates more energy features representing the specific character of the microbiota in the carious region than in previous works.

From a methodological point of view, all of the spectra presented in the work were obtained at the same wavelength of excitation (532 nm) and radiation power of 30 mW. To register signal of Raman scattering, the objective lens 60× was applied by compressing the initial radiating laser beam into the spot focused at the sample, with a diameter of 2 μm. The use of the proposed scheme and accumulation of the spectral signal from the microregion allowed us to register Raman spectra. However, in this case, we faced a photobleaching effect possibly caused by porphyrin degradation. In the case of observing luminescence spectra, less registration time was required. As a result, porphyrins that are present in the microregion were not subjected to the long-term impact of laser irradiation. This made it possible to confirm their presence in the microregion near carious lesion. For the large exposure time to the laser irradiation, in the case of Raman spectrum registration, porphyrin degradation occurred. This can be used to improve screening techniques.

In the laboratory conditions in vivo, it was frequently impossible to detect incipient noncavitated caries on the surface of tooth enamel, even with scanning electron microscopy and energy-dispersive X-ray microanalysis [35]. At the same time, the screening techniques applied in clinics sometimes suffer a lack of selectivity with respect to the separation of the intact and infected tissues, as sensitivity and reliability are needed for convenient and routine instruments for the practical diagnostics of the dental status of teeth under external inspection [30,33]. In our opinion, the optimal approach is to apply the diagnostic approaches based on the analysis of information about light-scattering (the use of Raman spectroscopy in vivo, as a diagnostic aid for discovering incipient caries [70,71,72]) and autofluorescent properties of the intact and affected dental tissue. Al-Obaidi et al. [52] have shown that comparing the data from the fluorescence spectra (shift of the signal to the red spectral range) and confocal Raman microscopy (determination of the value for the ratio of the Raman signal for the mineral phosphate band 𝜈_2_ at ~430 cm^−1^ and the organic matrix at ~2931 cm^−1^) enabled understanding of the changes in the chemical composition of the enamel and the separation of the demineralised and intact parts of the enamel on the surface of tooth enamel. However, until now there have not been any studies where both laser-induced fluorescence and microcombination scattering are used to detect and visualise incipient caries with the formation of microcavities in the deeper layers of enamel with a high spatial resolution.

## 5. Conclusions

The results of our pilot work suggest that the combination of the data obtained using LICV and microRaman scattering could allow the detection of not only incipient stages of caries in the enamel but also the areas associated with bacterial lesions. The use of several independent parameters obtained by combining and simultaneously analysing dental tissue with different techniques could reduce the number of missed lesions. This could also enable the unambiguous determination of a precise diagnosis related to the initial lesion (incipient caries) in the enamel without the use of excess X-ray irradiation. The data obtained in our pilot study have the potential to develop an effective preventive medical diagnostic approach. As a result, further personalised medical treatment can be specified.

## 6. Limitations

This study has certain limitations connected with the number of samples and the spatial resolution of the diagnostic method (microRaman spectroscopy) that are applied for the analysis of the features related to the molecular and energetic structure of biological tissues.

## Figures and Tables

**Table 1:** Description of the samples studied and their classification.

Sample Group/Number of Typical Samples, n	Description	ICDAS [53]
A n = 5	First molar tooth in the lower jaw: apparent carious lesion of dentin with destruction of dentin and enamel	6
B n = 5	Second molar in the lower jaw: carious lesion of enamel and dentin with destruction	5
C n = 5	Third molar in the upper jaw: destructive carious lesion of enamel and carious infection of dentin	4–3
D n = 5	Third molar in the upper jaw: demineralisation of the lateral wall of the fissure, developing caries of dentin, not detectable by visual examination	2
E n = 5	Third molar in the upper jaw: demineralisation of the lateral wall of the fissure, incipient caries in the fissure, not detectable by visual examination	1

## Data Availability

The data that support the findings of this study are available from the corresponding author upon reasonable request.
